# Comparative epidemiology, phylogenetics, and transmission patterns of severe influenza A/H3N2 in Australia from 2003 to 2017

**DOI:** 10.1111/irv.12772

**Published:** 2020-06-17

**Authors:** Jing Xia, Dillon C. Adam, Aye Moa, Abrar A. Chughtai, Ian G. Barr, Naomi Komadina, C. Raina MacIntyre

**Affiliations:** ^1^ Biosecurity Program Kirby Institute University of New South Wales Sydney NSW Australia; ^2^ College of Veterinary Medicine Sichuan Agricultural University Chengdu China; ^3^ School of Public Health and Community Medicine University of New South Wales Sydney NSW Australia; ^4^ WHO Collaborating Centre for Reference and Research on Influenza (VIDRL) Doherty Institute Melbourne Vic. Australia; ^5^ Department of Microbiology and Immunology Doherty Institute University of Melbourne Melbourne Vic. Australia; ^6^ School of Public Health and Preventive Medicine Monash University Melbourne Vic. Australia

**Keywords:** Australia, epidemiology, H3N2 subtype, influenza A virus, phylogeography, public health

## Abstract

**Background:**

Over the last two decades, Australia has experienced four severe influenza seasons caused by a predominance of influenza A (A/H3N2): 2003, 2007, 2012, and 2017.

**Methods:**

We compared the epidemiology, genetics, and transmission dynamics of severe A/H3N2 seasons in Australia from 2003 to 2017.

**Results:**

Since 2003, the proportion of notifications in 0‐4 years old has decreased, while it has increased in the age group >80 years old (*P* < .001). The genetic diversity of circulating influenza A/H3N2 viruses has also increased over time with the number of single nucleotide polymorphisms significantly (*P* < .05) increasing. We also identified five residue positions within or near the receptor binding site of HA (144, 145, 159, 189, and 225) undergoing frequent mutations that are likely involved in significant antigenic drift and possibly severity. The Australian state of Victoria was identified as a frequent location for transmission either to or from other states and territories over the study years. The states of New South Wales and Queensland were also frequently implicated as locations of transmission to other states and territories but less so over the years. This indicates a stable but also changing dynamic of A/H3N2 circulation in Australia.

**Conclusion:**

These results have important implications for future influenza surveillance and control policy in the country. Reasons for the change in age‐specific infection and increased genetic diversity of A/H3N2 viruses in recent years should be explored.

## 1 INTRODUCTION

Influenza is a common, highly infectious virus that spreads from person‐to‐person by droplet and airborne routes. Influenza A/H3N2 is one of four subtypes that circulate seasonally in humans every year causing annual epidemics in both temperate and tropical regions; the others being influenza A H1N1pdm09 (A/H1N1pdm09) and influenza B Yamagata (B/Yam) and Victoria (B/Vic).^1^ The predicted severity of seasonal influenza is not well understood but often depends on the predominate subtype in the circulation, vaccination coverage in the population, and the individual immune response of infected persons.^2^ Studies have frequently observed higher rates of hospitalization and reduced vaccine effectiveness among A/H3N2 predominate seasons when compared to seasonal A/H1N1pdm09 (and the pre‐pandemic seasonal A/H1N1) and influenza B viruses.^3^, ^4^, ^5^ Increased disease burden and mortality in elderly people and children have also been associated with A/H3N2 infection compared with other seasonal strains,^6^, ^7^ while influenza B typically causes disproportionate morbidity and mortality in children.^8^

Over the last 15 years, Australia has experienced four severe A/H3N2 influenza seasons: 2003, 2007, 2012, and 2017. Among these 2017 was the most severe, with reported vaccine effectiveness (VE) of approximately 10% for the then A/Hong Kong/4801/2014 A/H3N2 vaccine strain in 2017.^9^ An overall 33% vaccine effectiveness was estimated during the 2017 season in Australia for all strains.^9^ Notably, the circulating Australian A/H3N2 virus from 2017 quickly spread to other countries and regions such as the United States and Europe during the following 2017/2018 northern hemisphere season causing similarly severe seasons.^9^, ^10^, ^11^

In this study, we aimed to review the epidemiology and phylogenetics of the four most recent A/H3N2 predominant seasons in Australia as well as explore dynamics and temporal trends of A/H3N2 transmission in Australia.

## 2 METHODS

### 2.1 Review of epidemiology

We searched the National Notifiable Diseases Surveillance System (NNDSS) for all laboratory‐confirmed influenza notifications across all states and territories in Australia between 2001 and 2017 including data on subtype and age.^12^ Age‐stratified notification rates (per 100 000 population) were calculated using respective population estimates during the study years. Differences in notification rates between age groups and study years were assessed using a chi‐square test as the proportion of the total notification rate per year. Crude age‐stratified hospitalization and mortality rates due to influenza (per 100 000 population) between 2001 and 2017 were sourced from Australian Institute of Health and Welfare (AIHW) analysis of National Hospital Morbidity Database and National Mortality Database,^13^, ^14^, ^15^ respectively. Differences of those two rates and notification rate of laboratory‐confirmed influenza in each states/territory between severe and mild A/H3N2 seasons were also assessed using a chi‐square test. General practitioner surveillance reports on influenza‐like illness (ILI), seasonal antigenic drift, and VE were obtained from annual National Influenza Surveillance reports.^16^ Literature searches in Medline used a combination of keywords such as “vaccine effectiveness,” “influenza season,” “Australia,” “H3N2,” “vaccine,” “2003 season,” “2007 season,” “2012 season,” and “2017 season.”

### 2.2 Collection of sequence data

We searched GISAID for all human A/H3N2 hemagglutinin (HA) sequences collected during the study years with location and date of sampling metadata.^17^ The WHO Collaborating Centre for Reference and Research on Influenza supplied additional HA sequences for 2003 and 2007 that were not available in GISAID at the time. A total of 1619 HA sequences with the location (state or territory) and collection date were download from GISAID. We removed 223 records with duplicate isolate sources and egg‐isolate sequences leaving 1396 taxa. The number of sequences by states or territories is shown in Table S1. While most sequences were isolated in each state or territories capital city, for simplicity and subsequent analysis, we refer to each state/territory as a single discrete location.

To reduce the impact of spatial sampling bias across seasons, we randomly sampled up to 40 sequences from Australian states and territories with greater than 40 sequences per seasons while maintaining all sequences from states and territories with less than 40 sequences per season. A total of 87 selected HA sequences in 2003, 66 in 2007, 119 in 2012, and 310 in 2017 were included for subsequent phylogenetic analysis (Table S1). We further collected corresponding vaccine strains in GISAD for each study season: 2003 season (A/Panama/2007/99), 2007 season (A/Wisconsin/67/2005), 2012 season (A/Perth/16/2009), and 2017 season (A/Hong Kong/4801/2014).^18^

### 2.3 Phylogenetics analysis

We aligned each seasons taxa against the corresponding vaccine strain using MUSCLEv3.8.4^19^ and identified the proportion of unique mutations by subclade using Geneious v11.1.4. We measured the genetic diversity across each study season as the number of Single Nucleotide Polymorphisms (SNPs) per season using SeqMan via DNAstar Lasergene v7.1.^20^ To control for differences in sample size by season, the average number of SNPs per season was calculated across three randomly subsampled subsets of 50 sequences each. We used an non‐parametric Kruskal‐Wallis H test in ibm ^®^ spss Statistics 24 to determine significant differences in SNP counts of four seasons.^21^ We estimated selection ratios (d*N*/d*S*) for each season using three separate methods: Single likelihood ancestor counting (SLAC) and fixed‐effect likelihood (FEL) implemented via Datamonkey and default settings,^22^ and Bayesian renaissance counting (BRC) implemented in beastv1.8.4. In BEAST, we specified 100 million Markov Chain Monte Carlo generations sampling every 10 000 steps. We considered selected sites as significant if they were supported by at least two of the three methods used.

### 2.4 Phylogeography analysis

We generated time‐scaled phylogenetic trees for each A/H3N2 season using beast v1.8.4^23^, ^24^, ^25^ specifying a GTR + I + Γ4 nucleotide substitution model as determined using jModelTest.^26^ We selected a relaxed (uncorrelated log‐normal prior) molecular clock model over a strict clock model after additional model testing using path sampling (PS) and stepping‐stone sampling (SSS) methods in beast.v1.8.4 (Table S2), and a non‐parametric Bayesian skyline demographic tree prior.^27^, ^28^, ^29^, ^30^ We specified a symmetric discrete‐trait model using the Bayesian stochastic search variable selection (BSSVS) framework in beast.v1.8.4 to estimate transmission between each pair‐wise location per season.^23^, ^24^, ^25^ For each season, we again specified MCMC chains of 100 million generations sampling every 10 000 steps. We assessed for sufficient mixing and convergence using tracer.v1.6 after considering the first 10% of samples as burn‐in.^31^ We generated Maximum clade credibility (MCC) trees using treeannotator.v1.8.4^32^ and visualized each using figtree.v1.4.3.^33^ We used spread3.v0.9.6 to visualize transmission and the calculate Bayes factor (BF) support for each route.^34^ We considered statistical support for transmission as BF >3 which is convention in phylogeography studies.^35^

## 3 RESULTS

### 3.1 Epidemiology of A/H3N2 predominant seasons in year 2003, 2007, 2012, and 2017

Overall, a total of 714 867 laboratory‐confirmed notifications of influenza (type A, type B, and un‐typed) were reported to NNDSS in Australia between 2001 and 2017. Of those confirmed as influenza A, only a small proportion was subtyped with A/H3N2 accounting for 47 491 (6.64% = 47 491/714 867). The proportion of A/H3N2 in type A notifications was 38.48% (N = 47 491/123 425, excluding un‐subtyped A). A/H3N2 predominated in the years 2002‐2007, 2012, 2016, and 2017, with severe influenza seasons observed in 2003, 2007, 2012, and 2017 (Table S3). The proportion of A/H3N2 reported varied across these four seasons often co‐circulating with other type A and B viruses.

During severe seasons (2003, 2007, 2012, and 2017) A/H3N2 accounted for on average 82.06% (N = 4841/5899) of typed strains compared with 65.31% (N = 2001/3079) during mild A/H3N2 seasons (Table S3). There was no observable trend in increasing notification rates between states and territories when comparing severe and mild seasons (*P* > .05); however, the average notification rate of each state/territory was 1.75‐5.53 times higher compared with mild seasons (Table S4).

### 3.2 Influenza activity, ILI, and antigenic drift

**FIGURE 1:**
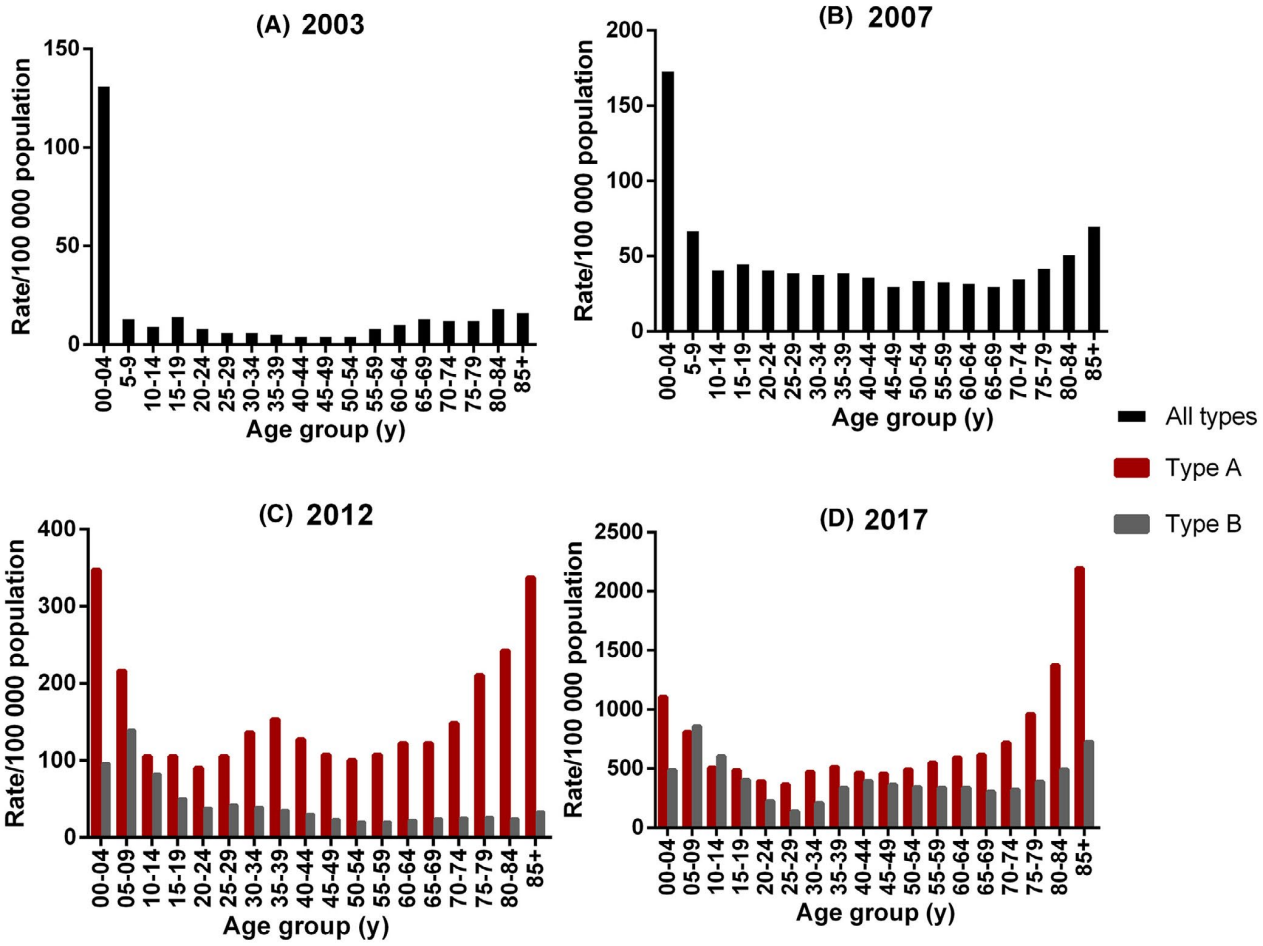
Notification rate of laboratory‐confirmed influenza (per 100 000 population), Australia, by 5‐year age group. A: 2003. B: 2007. C: 2012. D: 2017. Black bars represent the total number of influenza notifications by age group while red bars represent type A notifications, and grey bars represent type B notifications. The separate notification rate of type A and type B were not available in 2003 and 2007 seasons. Data source: NNDSS

Table S5 shows a descriptive summary of each seasons by influenza activity, influenza‐like illness (ILI), and antigenic drift. Overall, peak ILI activity has shifted from August to June (2003 to 2017) and weekly influenza positivity, defined as greater than 50% of ILI cases presenting to GPs who test positive for influenza per week, has increased from 5 to 6 weeks (2012 to 2017; 2003 and 2007 data not available). In contrast, peak ILI activity during previous mild A/H3N2 predominant seasons (2002, 2005, 2006, and 2016) occurred in mid‐late August with weekly Influenza positivity exceeding 50% for 3 weeks.^36^, ^37^, ^38^ During mild seasons when A/H3N2 was not predominant (2010, 2013, and 2015) peak ILI activity similarly occurred in mid‐late August with weekly Influenza positivity exceeding 50% for 2 weeks.^38^ Notification rates by age group have also significantly (*P* < .001) changed between the study years: The proportion of notifications in 0‐4 years old decreased, while increasing notifications in >80‐year‐olds since 2003 was observed (Figure 1 and Table S5), although this difference was not significant within the study years 2007 (*P* = .134), 2012 (*P* = .784), and 2017 (*P* = .470).

Proportion of positive patients at sentinel hospitals who were admitted to intensive care units (ICU) in severe seasons (8.9% in 2017, and 9% in 2012) were similar to mild A/H3N2 (10% in 2016) and non‐predominant seasons (14.2% in 2013 and 8.7% in 2015) (Table S5). There was also no significant difference of hospitalization rate and mortality rates between severe and mild seasons (*P* > .05). Pediatric influenza outcomes in severe seasons seemed similar to those observed in mild seasons (Table S6). However, the average crude hospitalization rates (5.91 times) and crude mortality rates (3.11 times) of elderly people were much higher compared with mild seasons (Table S6).

### 3.3 Phylogenetic analysis

**FIGURE 2:**
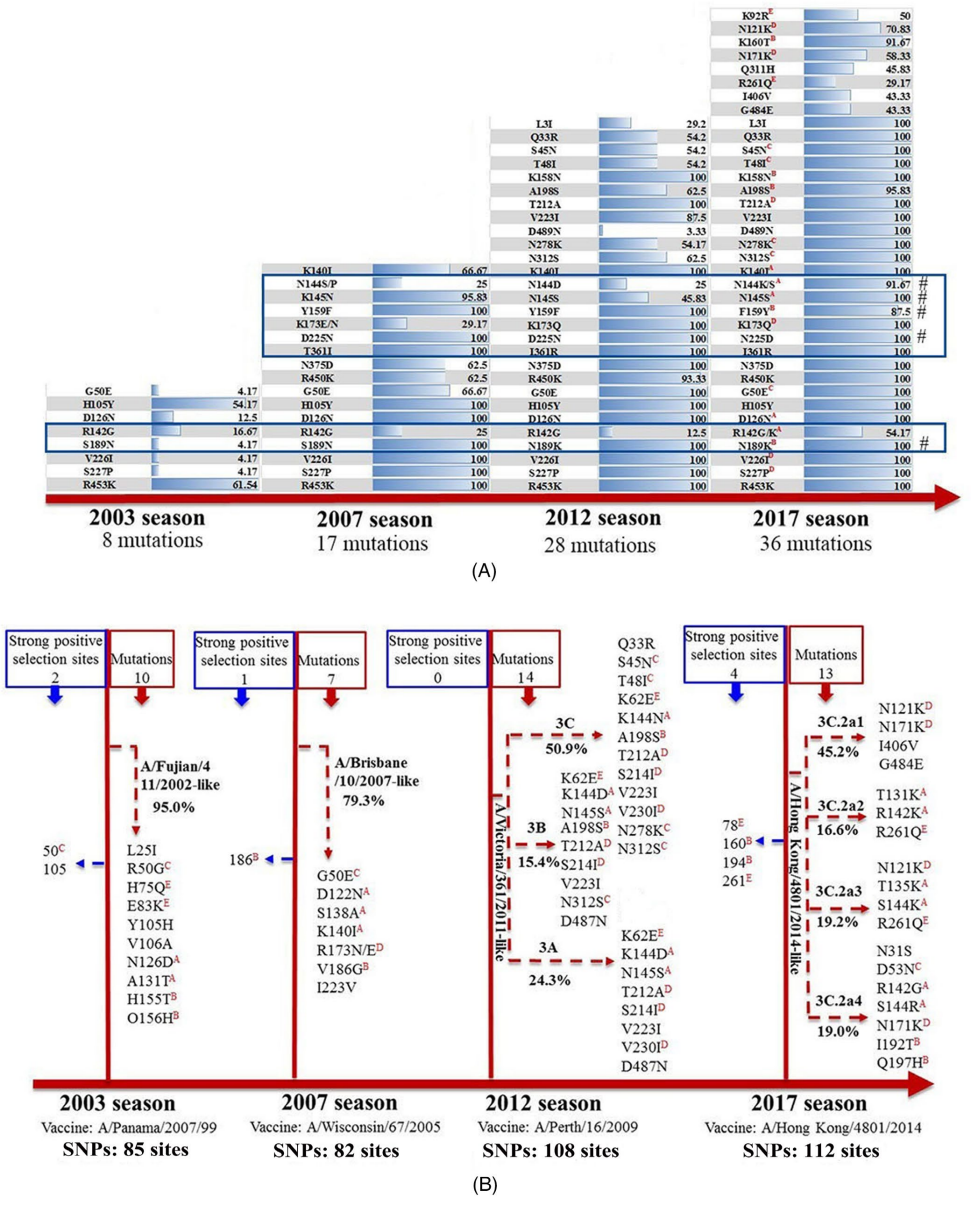
A, Timeline of mutations by season. Blue bars represent the proportion among taxa per season and red superscript represents the relative HA epitope. Blue boxes highlight sites that mutated at least twice and # represents the sites located on RBS and edge of RBS of HA protein. B, Mutations relative to vaccine‐strains (red dotted arrow) and HA sites under strong positive selection by season (blue dotted arrow). Percentage represents the proportion of taxa within that subclade each season

Figure 2 compares HA residue changes from 2003 to 2017 during the study years. From 2003 to 2017, a total of 36 mutations (29 in HA1 and seven in HA2) were observed in the HA surface protein (Figure 2A). Of those, 63.89% (23/36) of mutations were located within the known epitopes of HA.^39^ Residue changes at eight positions were reported in at least two seasons between 2003 and 2017, and five of those eight positions (144, 145, 159, 189, and 225) were located within the viral Receptor Binding Site (RBS) or edge of RBS (Figure 2A). Figure 2B shows that the genetic diversity of A/H3N2 virus has significantly increased (*P* < .05) across the four severe A/H3N2 seasons: A total of 85, 82, 108, and 112 SNPs relative to the corresponding study year's vaccine strain were identified in HA1 in 2003, 2007, 2012, and 2017 seasons, respectively.

**FIGURE 3:**
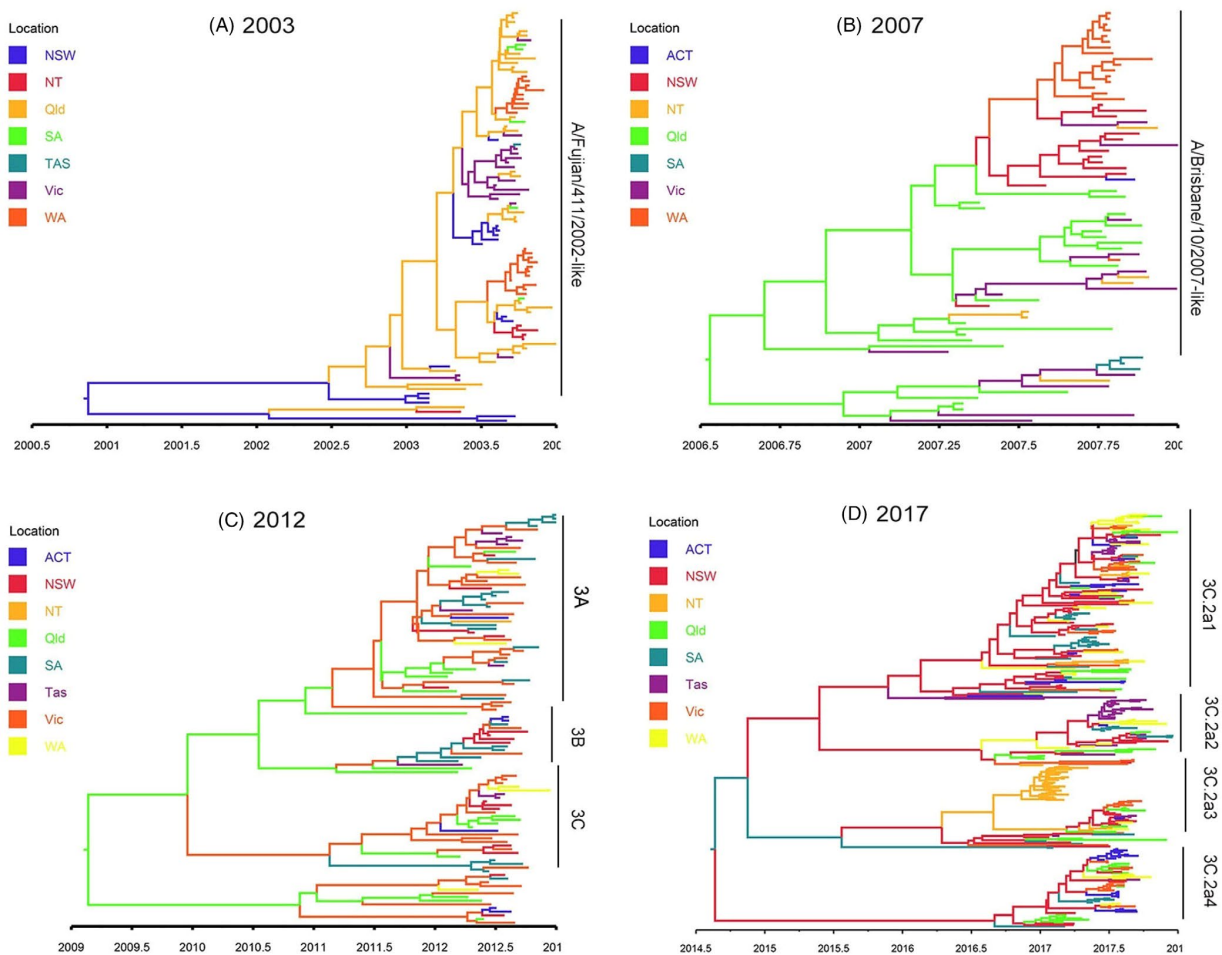
HA maximum clade credibility (MCC) phylogeographic tree of 2003, 2007, 2012, and 2017 A/H3N2 seasons. Branches were colored according to the most probable locations at which the nodes were formed. A, 2003 season, B, 2007 season, C, 2012 season, D, 2017 season

In 2017, the majority of A/H3N2 viruses circulating were closely related to the vaccine strain (A/Hong Kong/4801/2014) from clade 3C.2a,^40^ however, these then diversified into additional subclades: 3C.2a1, 3C.2a2, 3C.2a3, and 3C.2a4 (Figures 2B and 3D, *nextstrain nomenclature* ^41^). Notably, subclades within 3C.2a4 carried an additional seven substitutions (N31S, D53N, R142G, S144R, N171K, I192T, and Q197H). Of those, six substitutions were located across four epitopes, R142G and S144R in epitope A, I192T and Q197H in epitope B, D53N in epitope C and N171K in epitope D.

Across the other three severe A/H3N2 seasons, 2012 had the most (N = 14) mutations relative to the vaccine strain recommended for that year (Figure 2B) and isolates fell into three phylogenetic clades (3A, 3B, and 3C) (Figure 3C). Clade 3C viruses became the majority clade by 2012. Ten and seven substitutions were observed in the new subclades in 2003 and 2017 season, respectively, compared with the corresponding vaccine strain. HA positions under positive selection changed between each severe season (Table S7). We identified four positions in 2017 (residues 78, 160, 194, and 261), one in 2007 (reside 186) and two in 2003 (residues 50 and 105) exhibiting strong positive selection. No positions in 2012 were identified as being under positive selection.

### 3.4 Phylogeographic analysis of A/H3N2

**FIGURE 4:**
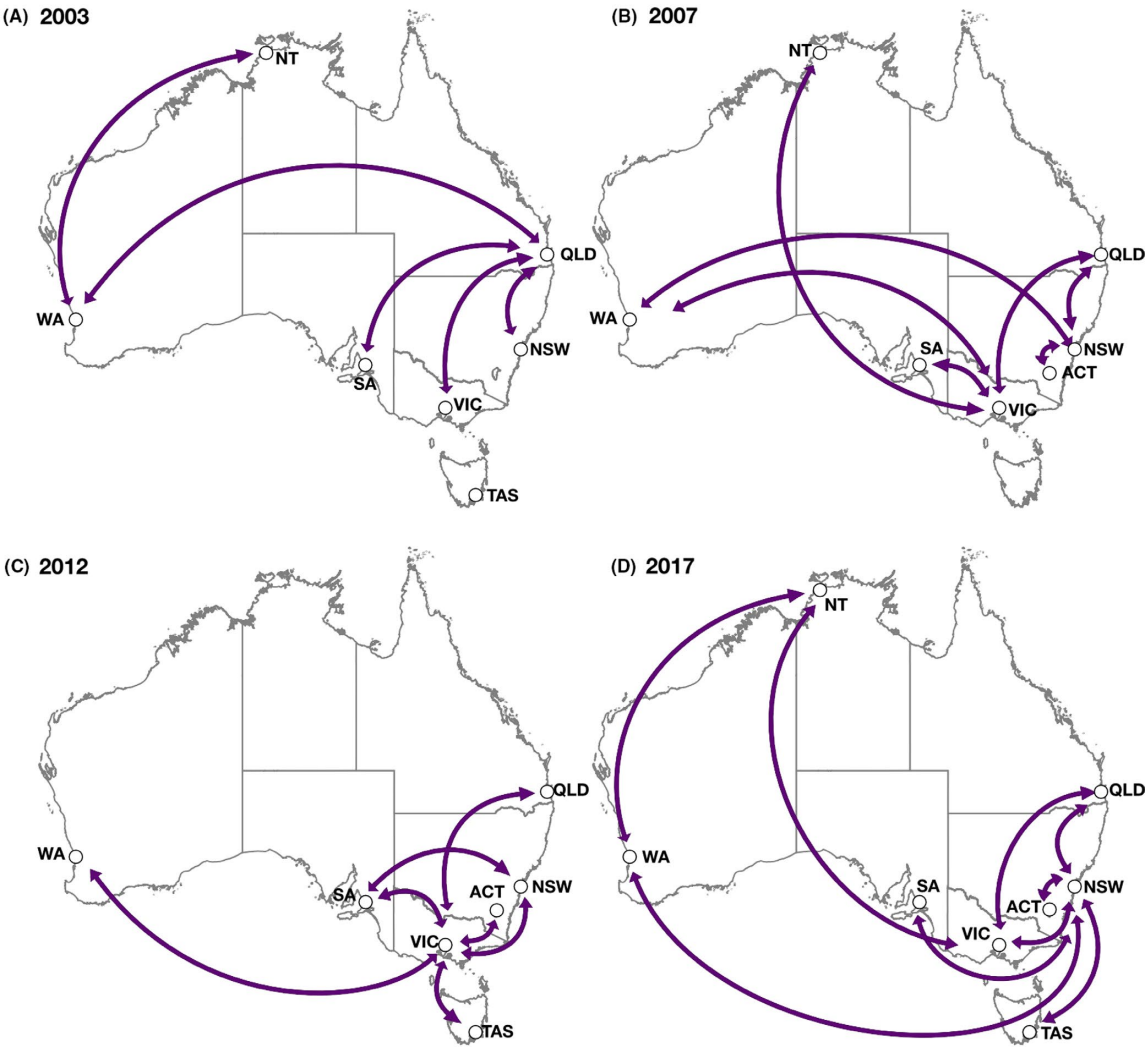
Phylogeographic projection of supported routes of influenza A/H3N2 transmission between states and territories in based on HA across four predominant H3 seasons

Between the eight states and territories of Australia across four A/H3N2 predominant seasons (2003, 2007, 2012, and 2017), we identified 28 routes (56 given two locations per route) of statistically supported (BF > 3) transmission, 11 of which were considered definitively supported (BF > 100) or 22 given two locations per definitive symmetric route (Figure 4 and Table S8). Across all four seasons, Victoria was the most frequently implicated location for transmission either to or from another state or territory (25%; N = 14/56) followed by New South Wales (21%; N = 12/56), and then Queensland (18%; N = 10/56). Victoria was again the most frequently supported location for transmission either to or from another state or territory among definitively supported routes (32%; N = 7/22) followed this time by Queensland (23%; N = 5/22) and then New South Wales (18%; N = 4/22). Transmission between Queensland and Victoria was supported in every season separately while transmission between Queensland and New South Wales was supported in three seasons excluding 2012. Most (50%; N = 14/28) other transmission pairs were supported in at least two other seasons while seven (25%; N = 7/28) remaining transmission pairs were only supported during a single season (Table S8).

## 4 DISCUSSION

In this study, we have reviewed the epidemiology of severe and mild A/H3N2 predominant seasons in Australia since 2003 and investigated the genetics and transmission patterns of four severe A/H3N2 seasons. We have observed a significant difference (*P* < .001) between age‐stratified notification rates across the study years, with increasing rates among those 80 years and over and decreasing rates among 0‐4 years (Figure 1). This suggests the elderly are an increasingly important risk group for A/H3N2, perhaps even more so than currently expected, as Australia's population continues to age: The proportion of over 80 population was 3.31%, 3.61%, 3.82%, and 3.91% in 2003, 2007, 2012, and 2017, respectively.^42^, ^43^, ^44^, ^45^ Recent increases in influenza vaccine coverage for children (children >6 months of age), from 10.1% in 2014 to approximately 30% in 2017 season may also explain this trend.^46^, ^47^ Influenza A/H3N2 is typically characterized by the limited circulating diversity of HA such that entire lineages are replaced within 2‐8 years.^48^ In our study, however, we observed that the genetic diversity of A/H3N2 per season has increased significantly (measured as the average number of SNPs per season) even when controlling for increased sampling effects (Figure 2B). This may be due to changing population dynamics rather than inherent viral changes and hypothesize that increasing global population size may provide the potential for increased diversity of globally circulating A/H3N2. Further in Australia, increased arrivals from international visitors and Australian residents traveling overseas similarly increasing opportunities for importation and spread of diverse global strains. These results have important implications on vaccination policy, where increasing circulating diversity means vaccination may have progressively lower impact if the vaccine cannot cover the increased diversity adequately.^49^ Alternatively, viral changes might also explain these changes. When counting the number of significant residue changes since 2003, we identified five positions, 144, 145, 159, 189, and 225, that were common in at least two severe seasons between 2003 and 2017. These positions were also located within the RBS or edge of RBS implicating their important role as potential determinates of antigenicity. In fact, four of the five positions have previously been identified as critical antigenic positions and similar amino acid changes have also been observed in other influenza subtypes.^50^, ^51^ Furthermore, frequent mutations at site 225, suspected to have a maintenance role in amantadine resistance, have been found among clusters of M2 characteristic A/H3N2 which bear S31N.^50^

Our study has also identified patterns and possible transmission trends in circulating A/H3N2 between states and territories in Australia. In each of the study years, we found strong evidence consistently linking transmission between Victoria and other states and territories across the continent. As cold temperatures are known to affect the transmissibility of influenza,^52^ this could be related to the cool‐temperate weather the state experiences during winter, along with a large growing population.^26^ New South Wales, Australia's most populous state, was also frequently implicated in transmission to or from other states and territories, and experiences similarly but less severe cool‐temperate winters. International tourism and Australian residents returning from overseas may also explain the observed results, with 63% of international overseas arrivals intending to stay in New South Wales and Victoria,^53^ and 62% of all short‐term Australian departures and arrivals coming from the same states^54^ increasing the likelihood of importation and ignition of seasonal outbreaks from these states, especially in the cities of Sydney (New South Wales) and Melbourne (Victoria). Interestingly, Queensland, a state which experiences rather mild winters and subtropical climates, was also frequently implicated in transmission across the study years. Potential reasons for this observation warrant further investigations but suggest demographic factors are more impactful on influenza transmission between Australian states and territories than climactic ones. Recent evidence of synchronization of seasonal onset across the continent also supports this conclusion.^55^ There was also an apparent trend towards increasing circulation on the east coast of Australia from 2003 to 2017. As Australia's population has continued to grow, particularly concentrated in major cities along the east coast,^26^ along with increasing domestic travel^28^ this is not unexpected but does highlight opportunities for epidemic control. For example, the emergence of a severe novel strain in Victoria or New South Wales might be expected to reach most major cities along the east coast in a relatively short period, perhaps weeks,^55^ and epidemic/pandemic plans should therefore account for this potential scenario.

There are a few of limitations to our study. We can only include data on laboratory‐confirmed influenza notifications to indicate the impact of A/H3N2 viruses. These notifications are likely to be a significant underestimate of the true incidence of influenza infection in the population.^56^ Furthermore, large numbers of un‐subtyped notifications also likely conceal the true proportion of subtype A/H3N2 among typed influenza A. We therefore make the assumption that the typed sample is representative of the true composition of circulating influenza A. The relative lack of sequence data in the earlier seasons studied here also means our analysis is likely to be affected by sampling bias, even when controlling for the known effects of oversampled locations in phylogeography studies.^57^ More frequent subtyping of influenza‐positive cases as well ensuring sequence data is routinely collected and uploaded onto publicly accessible databases would help to improve the accuracy of influenza surveillance in Australia and understandings of A/H3N2 severity.

## 5 CONCLUSION

We identify increasing rates of influenza notifications among those aged over 80 years in Australia as proportion of the total notification rate, while rates for those under 5 years have decreased as a proportion. This indicates an increasing influenza‐associated health burden for elderly Australians and may correlate with Australia's aging population. The genetic diversity of circulating A/H3N2 viruses has also increased since 2003 with more diversified strains observed in recent severe seasons. We hypothesize that increasing global population size and travel to Australia provides the potential for increased A/H3N2 circulating diversity, importation, and reduced VE, which has implications for domestic and international influenza vaccination policy. This, however, warrants further investigation. Lastly, we identified the states of Victoria and New South Wales as important locations for the dissemination of A/H3N2 viruses to other states and territories, possibly due to climate and/or population effects. Improving testing procedures to include routine subtyping and sequencing should be a priority for improving the accuracy and detailed data from influenza surveillance in Australia and enable a better understanding of the true burden of disease due to A/H3N2 influenza viruses.

## AUTHOR CONTRIBUTION

**Jing Xia:** Data curation (lead); Formal analysis (lead); Investigation (lead); Validation (supporting); Visualization (equal); Writing‐original draft (lead). **Dillon C. Adam:** Formal analysis (supporting); Investigation (supporting); Methodology (supporting); Supervision (supporting); Validation (lead); Visualization (equal); Writing‐original draft (supporting); Writing‐review & editing (supporting). **Aye Moa:** Methodology (supporting); Project administration (lead); Supervision (supporting); Writing‐review & editing (equal). **Abrar A. Chughtai:** Methodology (supporting); Supervision (supporting); Writing‐review & editing (equal). **Ian G. Barr:** Data curation (supporting); Methodology (supporting); Writing‐review & editing (supporting). **Naomi Komadina:** Data curation (supporting); Writing‐review & editing (supporting). **C. Raina MacIntyre:** Conceptualization (lead); Funding acquisition (lead); Methodology (lead); Project administration (supporting); Resources (lead); Supervision (lead); Writing‐review & editing (lead). 

## Supporting information

Table S1‐S8
