# Supplementary material for: Comparative epidemiology, phylogenetics, and transmission patterns of severe influenza A/H3N2 in Australia from 2003 to 2017
Source: Influenza Other Respir Viruses. 2020 Jun 17;14(6):700–9. doi: 10.1111/irv.12772 (PMC7578330; doi:10.1111/irv.12772)
Supplement: Supplementary file 1 — Table S1‐S8 [file IRV-14-700-s001.docx]

**Table S1.** Initial number of HA sequences isolated in Australia and Number of subsampled HA sequences using in phylogeography analysis in 2003, 2007, 2012, 2017, by state and territory.

| Year | ACT | NSW | NT | QLD | SA | TAS | VIC | WA | Total |
| --- | --- | --- | --- | --- | --- | --- | --- | --- | --- |
|  | (subsamples) | (subsamples) | (subsamples) | (subsamples) | (subsamples) | (subsamples) | (subsamples) | (subsamples) | (subsamples) |
| 2003 | -(-) | 14(14) | 5(5) | 27(27) | 5(5) | 1(1) | 15(15) | 20(20) | 87(87) |
| 2007 | 1(1) | 12(12) | 6(6) | 15(15) | 3(3) | -(-) | 12(12) | 17(17) | 66(66) |
| 2012 | 6(6) | 16(16) | 1(1) | 17(17) | 24(24) | 9(9) | 73(40) | 6(6) | 152(119) |
| 2017 | 33(33) | 258(40) | 37(37) | 127(40) | 203(40) | 72(40) | 313(40) | 48(40) | 1091(310) |
| Total | 40(40) | 300(82) | 49(49) | 186(99) | 235(72) | 82(50) | 413(107) | 91(83) | 1396(582) |

Data source: GISAID. The “-” represents no data. The (N) represents the number of subsampled HA sequences using in phylogeography analysis. Data before (N) represents the initial number of HA sequences.

**Table S2.** Features of models of H3N2 seasons in 2003, 2012, 2007 and 2017

| Influenza season | DNA substitution model | Molecular clock | PS | SS | Root heights  (HPD) | Mean substitution rate /per site  (HPD) |
| --- | --- | --- | --- | --- | --- | --- |
| 2017 | GTR+I+G4 | Relaxed | **-8717.0035** | **-8729.2662** | 2.74 [1.95, 3.57] | 4.51×10^-3^ [3.68×10^-3^, 5.37×10^-3^] |
|  | GTR+I+G4 | Strict | -8732.5153 | -8744.9151 | - | - |
| 2012 | GTR+I+G4 | Relaxed | **-5682.3178** | **-5686.1570** | 3.94 [2.79, 5.33] | 3.20×10^-3^ [2.27×10^-3^, 4.09×10^-3^] |
|  | GTR+I+G4 | Strict | -5686.5440 | -5689.7887 | - | - |
| 2007 | **GTR+I+G4** | **Relaxed** | **-4048.3042** | **-4022.7873** | 1.51 [1.10, 1.99] | 9.92×10^-3^ [6.44×10^-3^, 1.37×10^-2^] |
|  | GTR+I+G4 | Strict | -4097.0400 | -4079.5088 | - | - |
| 2003 | GTR+I+G4 | Relaxed | **-2999.3567** | **-2850.7436** | 3.21 [1.74, 4.89] | 8.13×10^-3^ [4.88×10^-3^, 1.14×10^-2^] |
|  | GTR+I+G4 | Strict | -3467.4722 | -3467.7859 | - | - |
| Average |  |  |  |  | 2.85 | 6.44×10^-3^ |

The molecular clock model with higher value of PS and SS was considered the best-fit model (black bold). Note: HPD is the 95% highest posterior density interval.

**Table S3**: Laboratory-confirmed influenza notifications and proportion by type and subtype, Australia, severe (2003, 2007, 2012 and 2017) and mild H3N2 seasons.

|  | Year | Notifications subtype / un-subtype A | | | Notifications type A (%)¶ | Notifications type B (%)¶ | Notifications others/untyped (%)¶ | Total notifications |
| --- | --- | --- | --- | --- | --- | --- | --- | --- |
|  |  | H3N2 (%)* | H1N1/H1N1 pdm09 (%)* | Un-subtyped (%)* |  |  |  |  |
| Severe H3N2 seasons | 2003 | 315 (99.06) | 3 (0.94) | 2,811 | 3,129 (89.66) | 124 (3.55) | 237 (6.79) | 3,490 |
|  | 2007 | 885 (66.49) | 446 (33.51) | 7,902 | 9,233 (87.29) | 956 (9.04) | 388 (3.67) | 10,577 |
|  | 2012 | 7,340 (95.95) | 310 (4.05) | 26,258 | 33,908 (76.16) | 10,538 (23.67) | 78 (0.18) | 44,524 |
|  | 2017 | 10,825 (75.68) | 3,475 (24.31) | 140,133 | 154,433 (61.58) | 95,586 (38.12) | 764 (0.30) | 250,783 |
|  | **Average** | **4,841 (82.06)** | **1,058 (17.94)** | **44,276** | **50,175 (64.87)** | **26,801 (34.65)** | **366 (0.47)** | **77,343** |
| Mild H3N2 seasons | 2002 | 281 (99.65) | 1 (0.35) | 2,399 | 2,681 (73.25) | 868 (23.72) | 111 (3.03) | 3,660 |
|  | 2004 | 35 (94.59) | 2 (5.41) | 1,538 | 1,575 (76.72) | 370 (18.02) | 108 (5.26) | 2,053 |
|  | 2005 | 75 (75.00) | 25 (25.00) | 3,308 | 3,408 (74.46) | 1,000 (21.85) | 169 (3.69) | 4,577 |
|  | 2006 | 123 (90.44) | 13 (9.56) | 2,207 | 2,343 (70.57) | 877 (26.42) | 100 (3.01) | 3,320 |
|  | 2016‽ | 9,494 (63.97) | 5,348 (36.03) | 64,693 | 79,535 (89.12) | 9,594 (10.75) | 119 (0.13) | 89,248 |
|  | **Average** | **2,001 (64.99)** | **1,078 (35.01)** | **14,829** | **17,908 (87.05)** | **2,542 (12.36)** | **121 (0.59)** | **20,572** |

Data source: NNDSS. ¶ represents the proportion of notifications type A or B or others / total notifications within the year. * represents the proportion of notifications subtype H3N2 (or H1N1)/ notifications subtyped A. ‽ represents the influenza notifications from Australian Capital Territory (ACT) were not included in year 2016.

**Table S4**. Notification rate of laboratory confirmed influenza (per 100,000 population), Australia, in severe and mild H3N2 influenza seasons, by state and territory.

|  | Year | ACT | NSW | NT | QLD | SA | TAS | VIC | WA | Australia |
| --- | --- | --- | --- | --- | --- | --- | --- | --- | --- | --- |
| Severe H3N2 seasons | 2003 | 2.4 | 13 | 74.9 | 23.7 | 20.5 | 1.5 | 13.2 | 31.5 | 17.7 |
|  | 2007 | 114.1 | 30.1 | 85.6 | 111.3 | 18.1 | 84.1 | 30.9 | 49.3 | 50.6 |
|  | 2012 | 177.1 | 109.5 | 187.8 | 368.4 | 379.5 | 213.6 | 106.1 | 215.8 | 196 |
|  | 2017 | 751.9 | 1320.2 | 595.5 | 1148.5 | 1652.6 | 671.1 | 761.2 | 233.4 | 1021.1 |
|  | **Average** | **261.38** | **368.2** | **235.95** | **412.98** | **517.68** | **242.58** | **227.85** | **132.5** | **321.35** |
| Mild H3N2 seasons | 2002 | 5.9 | 15.4 | 26.2 | 31.3 | 19.3 | 1.5 | 12.3 | 28.2 | 18.8 |
|  | 2004 | 0.3 | 14.1 | 19.2 | 16.1 | 5 | 0.6 | 4.2 | 9.4 | 10.3 |
|  | 2005 | 12.7 | 21.1 | 29.6 | 43.2 | 18 | 3.9 | 11.9 | 23.1 | 22.6 |
|  | 2006 | 23.9 | 10 | 19.6 | 43.7 | 5.7 | 9.6 | 8.3 | 10.4 | 16.2 |
|  | 2016 | 397.9 | 460.4 | 285.3 | 480.5 | 459.6 | 203.9 | 209.4 | 306.6 | 375.7 |
|  | **Average** | **88.14** | **104.2** | **75.98** | **122.96** | **101.52** | **43.9** | **49.22** | **75.54** | **88.72** |
| Average Severe/Mild |  | 2.97 | 3.53 | 3.11 | 3.36 | 5.10 | 5.53 | 4.63 | 1.75 | 3.62 |

Data source: NNDSS. The underlined data represents the highest notification rate. Abbreviations: ACT, Australian Capital Territory; NSW, New South Wales; NT, Northern Territory; QLD, Queensland; SA, South Australia; TAS, Tasmania; VIC, Victoria; WA, Western Australia.

**Table S5.** Summary of Seasonal and Viral Characteristics of four severe H3N2 seasons in Australia 2003 – 2017

|  | **Seasonal characteristics** | **Viral characteristics** |
| --- | --- | --- |
| 2017  (H3N2, co-circulated with Influenza B) | ● The peak ILI activity started in mid-June (one month earlier).  ● Peak GP ILI consultation rate was about 25.55/per 1,000 ^1^.  ● 47% of patients with ILI tested positive for influenza and 8.9% influenza patients were admitted to ICU ^2^.  ● Weekly influenza positivity exceeded 50% for six weeks.  ● Pediatric influenza outcomes (ICU admission and case fatality rate) seem similar to those observed in previous years ^3^.  ● High risk age group : ≥ 80, 5-9 and 0-4 years old ^2^. | ● H3N2 Vaccine strain: A/Hong Kong/4801/2014, circulated strain: A/Hong Kong/4801/2014-like.  ● HAI testing of samples from GP indicated that isolates were generally antigenically like vaccine strains.  ●VE against H3N2 was 10% (95% CI: -16,31), for H1N1pdm09 was 50% (95% CI: 8,74) and for type B was 57% (95% CI: 41,69) ^4^. |
| 2012  (H3N2, co-circulated with Influenza B) | ● The peak in ILI activity around in mid-July, while mid-August in Queensland.  ● Peak GP ILI consultation rate was about 19.05/per 1,000 ^1^.  ● 45% of patients with ILI tested positive for influenza and 9% influenza patients were admitted to ICU ^5^.  ● Influenza positivity in patients presenting to sentinel GPs with ILI exceeded 50%: 5 weeks ^2^.  ● High risk age group: 0-4, ≥ 80, 5-9 and 30-44 years old ^5^. | ● H3 isolates were mostly genetically and antigenically distinguishable from the vaccine strain A/Perth/16/2009. Circulated strain: A/Victoria/361/2011-like.  ● The majority of influenza B viruses are similar to the strain in the current vaccine.  ● The adjusted VE estimate for any type of influenza was 45% (95% CI: 8, 66) and for H3N2 was 35% (95% CI:-11, 62) ^6^. |
| 2007  (H3N2, co-circulated with H1N1) | ● The ILI activity peaked in mid-July to mid-August.  ● Peak GP ILI consultation rate was about 44.96/per 1,000 (3 times the mean rate over the previous 5 years) ^1^.  ● High risk age group: 0-4, 5-9 and >80 years ^7^. | ● H3N2 Vaccine strain: A/Wisconsin/67/2005, newly emergent strain: A/Brisbane/10/2007-like.  ● H1N1 vaccine strain: A/New Caledonia/20/99, newly emergent strain: A/Brisbane/59/2007-like.  ● Antigenic drift away from the vaccine strain was observed with the H3N2 viruses and was also seen with most of A/H1N1 viruses ^7^.  ●Adjusted VE against GP presentation for H3N2 was 68% (95% CI, 15, 85), for A/H1N1 was 27% (95% CI, -92, 72) in Victoria ^8,9^. |
| 2003  (H3N2) | ● The peak ILI activity started in mid-August.  ● Peak GP ILI consultation rate was about 24/per 1,000.  ● High risk age group: 0-4 years ^10^. | ● H3N2 Vaccine strain: A/Panama/2007/99, circulated strain: A/Fujian/411/2002(H3N2)-like.  ● The HA1 sequence of prevalent virus was similar with A/Fujian/411/2002(H3N2)-like  ● The H3N2 virus have shown a significant antigenic drift.  ● The 2003 Australian influenza vaccine contained A/Panama/2007/99, which induced 2–4-fold lower antibody response against the drifted strain ^10,11^. |

Note: the activity of ILI from sentinel general practitioner surveillance systems referred to the annual report of the national influenza surveillance scheme of each year. The virology referred to the annual report and articles. The Average Peak GP ILI consultation rate of H3N2 predominate season

**Table S6**. Crude hospitalization rates and mortality rates due to influenza (per 100,000 population), Australia, in severe and mild H3N2 influenza seasons, by age groups.

|  |  | Crude hospitalization rates | | | Crude mortality rates | | | |
| --- | --- | --- | --- | --- | --- | --- | --- | --- |
|  | Year | 0-4 years | 5-64 years | 65 years and over | 0-4 years | 5-64 years | | 65 years and over |
| Severe H3N2 seasons | 2003 | 91.6 | 6.5 | 16.3 | 0.16 | | 0.06 | 2.11 |
|  | 2007 | 72.3 | 9.0 | 17.0 | 0.37 | | 0.08 | 2.01 |
|  | 2012 | 89.5 | 16.7 | 65.3 | 0.33 | | 0.09 | 4.08 |
|  | 2017 | 124 | 114 | 870 | 0.12 | | 0.4 | 31.05 |
|  | **Average** | **94.35^!^** | **36.55^!^** | **242.15^!^** | **0.25** | | **0.16** | **9.81** |
| Mild H3N2 seasons | 2002 | 69.9 | 7.4 | 17.2 | 0.08 | | 0.06 | 1.83 |
|  | 2004 | 27.7 | 3.6 | 9.3 | 0.24 | | 0.02 | 1.17 |
|  | 2005 | 38.5 | 6.3 | 11.1 | 0.16 | | 0.06 | 1.07 |
|  | 2006 | 28.9 | 3.8 | 6.3 | 0.23 | | 0.01 | 0.41 |
|  | 2016 | 91.8 | 25.0 | 161.1 | 0.19 | | 0.25 | 11.27 |
|  | **Average** | **51.36** | **9.22** | **41** | **0.18** | | **0.08** | **3.15** |
| Average Severe/Mild |  | 1.84 | 3.96 | 5.91 | 1.39 | | 2.00 | 3.11 |

Data source: Source of the crude mortality rates: Australian Institute of Health and Welfare (AIHW) analysis of National Mortality Database. Source of the crude hospitalization rates: AIHW analysis of National Hospital Morbidity Database. The “!” represents the crude hospitalization rates of influenza and pneumonia (vaccine-preventable), while most of hospitalizations were due to influenza in 2017. The underlined data represents the highest rate.

**Table S7.** Positive selected sites on the HA coding sequences of H3N2 isolated in Australia, 2003, 2007, 2012 and 2017.

| 2017 | | | |  | 2012 | | | |  | 2007 | | | |  | 2003 | | | |
| --- | --- | --- | --- | --- | --- | --- | --- | --- | --- | --- | --- | --- | --- | --- | --- | --- | --- | --- |
| Sites | Bayesian  Mean* | FEL | SLAC |  | Sites | Bayesian  Mean* | FEL | SLAC |  | Sites | Bayesian  Mean* | FEL | SLAC |  | Sites | Bayesian  Mean* | FEL | SLAC |
| 31 | 2.0877  (1.4534, 2.8233) |  |  |  | 45^C^ | 1.7674  (1.0097, 2.3447) | - | - |  | 6 | 2.493  (1.5406, 4.1240) |  | - |  | 25 | 1.7045  (1.00028, 2.75742) |  | - |
| **78^E^** | **2.5802**  **(1.7979, 3.3623)** | + |  |  | **53^C^** | **3.0473**  **(1.8447, 4.4552)** |  |  |  | 50^C^ | 2.3340  (1.0754, 3.9942) |  |  |  | 48^C^ | 1.69987  (1.00786,2.71214) |  |  |
| 122^A^ | 1.6092  (1.0775, 2.2026) |  |  |  | 145^A^ | 1.9198  (1.1897, 2.9921) |  |  |  | 140^A^ | 2.4774  (1.5255, 4.0076) |  |  |  | 50^C^ | 4.3463  (2.52042,7.08483) | **+** |  |
| 128^B^ | 1.5865  (1.1412, 2.1586) |  |  |  | 230^D^ | 1.8746  (1.1066, 2.7846) |  |  |  | 142^A^ | 2.8355  (1.5911, 5.0740) |  |  |  | 94^E^ | 1.72022  (1.00319,2.87094) |  |  |
| 142^A^ | 2.3104  (1.5117, 3.1817) |  |  |  | 418 | 1.77  (1.1188, 2.5112) |  |  |  | 144^A^ | 3.6374  (2.2113, 6.0681) |  |  |  | **105** | **4.35837**  **(2.50696,7.19813)** | + |  |
| 144^A^ | 2.2981  (1.3574, 3.5412) |  |  |  |  |  |  |  |  | 156^B^ | 3.5549  (2.1589, 5.8090) |  |  |  | 107 | 1.70449  (1.00261,2.78081) |  |  |
| **160^B^** | **5.5311**  **(3.9361, 7.5527)** | + | + |  |  |  |  |  |  | 173^D^ | 6.7414  (3.7835, 11.183) |  |  |  | 124^A^ | 3.02379  (1.74103,5.01358) |  |  |
| 171^D^ | 1.6094  (1.1096, 2.33) |  |  |  |  |  |  |  |  | **186^B^** | **4.8124**  **(2.8114, 8.2669)** | + |  |  | 128^B^ | 1.71117  (1.00565,2.7938) |  |  |
| 192^B^ | 1.6386  (1.1527, 2.3201) |  |  |  |  |  |  |  |  | 190^B^ | 2.4692  (1.5230, 4.0201) |  |  |  | 142^A^ | 1.70953  (1.00078,2.81756) |  |  |
| **194^B^** | **1.6618**  **(1.116, 2.1932)** | + |  |  |  |  |  |  |  | 264^E^ | 2.2785  (1.0963, 3.8374) |  |  |  | 179^D^ | 1.71104  (1.00078,2.78298) |  |  |
| 197^B^ | 2.0667  (1.1401, 2.9783) |  |  |  |  |  |  |  |  | 269 | 2.2642  (1.0919, 3.6910) |  |  |  | 193^B^ | 2.62012  (1.10671,4.79642) |  |  |
| 242^D^ | 1.6008  (1.0223, 2.1312) |  |  |  |  |  |  |  |  | 275^C^ | 2.4618  (1.5321, 3.9008) |  |  |  | 201^D^ | 3.45711  (1.81316,6.36973) |  |  |
| **261^E^** | **2.5856**  **(1.8351, 3.5539)** | + | + |  |  |  |  |  |  |  |  |  |  |  | 227^D^ | 1.71029  (1.00565,2.77135) |  |  |
| 431 | **-** | + |  |  |  |  |  |  |  |  |  |  |  |  | 252 | 1.71442  (1.0022,2.88244) |  |  |
| 311^C^ | 1.5811  (1.0873, 2.1556) |  |  |  |  |  |  |  |  |  |  |  |  |  | 262^E^ | 2.64228  (1.09591,5.72502) |  |  |
| 522 | 2.081  (1.3466, 2.79) |  |  |  |  |  |  |  |  |  |  |  |  |  | 299^C^ | 1.71159  (1.00078,2.80831) |  |  |

Black bold resides were supported as strong evidence under positive selection pressure. The “-” represents no positive selected sites under the method. The “+” represents positive selected at the same sites. Amino acid residue numbering was based on the HA1 of H3N2 vaccine strain A/Hong Kong/4801/2014. Superscript ^A, B, C, D, E^ represents the epitope. Mean is the mean dN/dS estimate. The * represents the 95% HPD (highest posterior density interval).

**Table S8.** Posterior probabilities and Bayes factor (>3) support for transmissions between discrete locations of A/H3N2 virus in Australia, in year 2003, 2007, 2012 and 2017

| Influenza Season | Location A | Location B | Bayes Factor | Posterior Probability |
| --- | --- | --- | --- | --- |
| 2003 | Qld | NSW | 4807.856 | 0.999556 |
|  | Qld | SA | 435.1346 | 0.995112 |
|  | Qld | Vic | 268.848 | 0.992112 |
|  | Qld | WA | 20.33907 | 0.904899 |
|  | Qld | NT | 4.054923 | 0.654816 |
| 2007 | Qld | Vic | 19237.84 | 1 |
|  | NT | Vic | 200.3885 | 0.989446 |
|  | SA | Vic | 27.92492 | 0.928897 |
|  | ACT | NSW | 27.10255 | 0.926897 |
|  | NSW | WA | 5.839235 | 0.73203 |
|  | Vic | WA | 5.592464 | 0.723475 |
|  | NSW | Qld | 3.867448 | 0.64404 |
| 2012 | SA | Vic | 23756.43 | 1 |
|  | NSW | Vic | 696.1564 | 0.996223 |
|  | Qld | Vic | 492.3409 | 0.994667 |
|  | Tas | Vic | 400.0564 | 0.993445 |
|  | Vic | WA | 26.98517 | 0.910899 |
|  | ACT | Vic | 6.751332 | 0.71892 |
|  | NSW | SA | 4.512434 | 0.63093 |
| 2017 | NSW | WA | 23756.43 | 0.999889 |
|  | NSW | SA | 421.6294 | 0.993778 |
|  | NSW | Vic | 56.90693 | 0.955672 |
|  | NSW | Qld | 52.10479 | 0.951783 |
|  | NSW | Tas | 41.03515 | 0.939562 |
|  | ACT | NSW | 40.79562 | 0.939229 |
|  | NT | WA | 24.63831 | 0.903233 |
|  | NT | Vic | 8.400855 | 0.760915 |
|  | Qld | Vic | 4.054977 | 0.60571 |

**References**

1. Australian Sentinel Practitioners Research Network (ASPREN). In.

2. 2017 Influenza Season in Australia, A summary from the National Influenza Surveillance Committee. In: Health Do, ed2017.

3. Blyth CC, Macartney KK, McRae J, et al. Influenza Epidemiology, Vaccine Coverage and Vaccine Effectiveness in Children Admitted to Sentinel Australian Hospitals in 2017: Results from the PAEDS-FluCAN Collaboration. *Clin Infect Dis.* 2019;68(6):940-948.

4. Sullivan SG, Chilver MB, Carville KS, et al. Low interim influenza vaccine effectiveness, Australia, 1 May to 24 September 2017. *Euro Surveill.* 2017;22(43).

5. Australian influenza report 2012—29 September to 12 October 2012. In: Health Do, ed2012.

6. Sullivan SG, Komadina N, Grant K, Jelley L, Papadakis G, Kelly H. Influenza vaccine effectiveness during the 2012 influenza season in Victoria, Australia: influences of waning immunity and vaccine match. *J Med Virol.* 2014;86(6):1017-1025.

7. Owen R, Barr IG, Pengilley A, Liu C, Paterson B, Kaczmarek M. Annual report of the National Influenza Surveillance Scheme, 2007. *Commun Dis Intell.* 2008;32(2):208-226.

8. Kelly HA, Sullivan SG, Grant KA, Fielding JE. Moderate influenza vaccine effectiveness with variable effectiveness by match between circulating and vaccine strains in Australian adults aged 20-64 years, 2007-2011. *Influenza Other Respir Viruses.* 2013;7(5):729-737.

9. Fielding JE, Grant KA, Papadakis G, Kelly HA. Estimation of type- and subtype-specific influenza vaccine effectiveness in Victoria, Australia using a test negative case control method, 2007-2008. *BMC Infect Dis.* 2011;11:170.

10. Yohannes K, Roche P, Hampson A, Miller M, Spencer J. Annual report of the National Influenza Surveillance Scheme, 2003. *Commun Dis Intell Q Rep.* 2004;28(2):160-168.

11. Barr IG, Komadina N, Hurt AC, et al. An influenza A(H3) reassortant was epidemic in Australia and New Zealand in 2003. *J Med Virol.* 2005;76(3):391-397.
